# Injuries and Fatalities Related to Freediving: A Case Report and Literature Review

**DOI:** 10.7759/cureus.30353

**Published:** 2022-10-16

**Authors:** Michael F Allen, Deborah E Allen

**Affiliations:** 1 Sports Medicine, Royal United Hospital, Bath, GBR; 2 Sports Medicine, Hull York Medical School, York, GBR

**Keywords:** glossopharyngeal insufflation, apnoea, breath-hold diving, freediving, watersports, pneumothorax

## Abstract

This case report and literature review aim to explore the range of injuries sustained in the sport of freediving.

The case report involves a 37-year-old patient who sustained a pneumothorax secondary to freediving. We conducted the literature review to analyse the injuries associated with freediving. We used the combination of search terms ‘freediving”, “injuries”, and “breath-hold diving” on the database PubMed®. A total of 40 studies were eligible for inclusion in this review. The search revealed a wide range of ophthalmological, pulmonary, neurological, ear, nose, and throat injuries, along with several fatalities.

Freediving is a sport performed in extreme environments and, if undertaken by inexperienced, untrained, or competition divers, can lead to severe injury or even death. However, the risk of damage can be reduced by performing it responsibly with the appropriate training and by using proper safety measures. Future research is warranted into the psychological, physiological, and economic benefits of freediving at both individual and community levels.

## Introduction

Freediving is currently growing in popularity as a recreational sport, but the discipline has been practised worldwide for centuries. It is unclear where the origins of the sport lie. However, different cultures have utilised the skill in different ways across the world. The ancient Greeks engaged in freediving to collect sponges from the seabed before the invention of scuba tanks [1]. Similarly, Japanese Ama divers, famously known as "diving women", used freediving to collect pearls and fish [2].

Today, the sport is becoming more prevalent as a recreational activity, an affordable alternative to scuba diving. Participants learn to improve self-discipline and breath-holding techniques and to improve the length of dives. Various disciplines exist within freediving, and these differ from each other based on the equipment used: fins, guide ropes, weights, or even lift bags are used to aid ascendance. Some individuals take the sport to extremes, braving freezing temperatures or tremendous depths. The current world records for Constant Weight with No Fins (CNF) are held by Alessia Zecchini and William Trubridge, who reached depths of 73m and 102m, respectively [3,4].

Freediving is becoming more commonplace, both commercially and in the military. Breath-hold divers can perform some simple tasks quickly and more efficiently than scuba divers. The skill of apnoea is also utilised within such environments to promote safety, such as in the surfing community [5]. The dangers of the sport are very clear: as divers descend, extreme pressures are exerted on their bodies. The further they travel from the surface, the more limited their options for escape, should something go wrong. Fatalities can occur when divers push themselves beyond their limits.

## Case presentation

A 37-year-old man presented to the emergency department with a five-day history of mild left-sided chest discomfort. He described the pain as a constant ache that was exacerbated by coughing. There were no associated symptoms such as shortness of breath, pain radiation or fever, or any alleviating factors. He stated that he engaged in freediving and reported that on his last dive, he had pushed himself further than in previous attempts, to reach a personal best. When he had surfaced, he started to notice the discomfort in his chest. He had taken simple analgesia and ignored the pain, hoping that it would get better. On the morning that he presented, he had gone for a short run, which had exacerbated the pain and left him shorter of breath than usual, and prompted him to present to the department. He had no previous medical history, and he was generally fit and well, apart from being an occasional smoker. On examination, he appeared well, and nothing remarkable was observed; he underwent a chest X-ray, which revealed a small left-sided pneumothorax. He was given safety netting advice and discharged with follow-up. A repeat chest x-ray after two weeks showed complete resolution of the pneumothorax.

## Discussion

This case focuses on a freediving-induced simple pneumothorax. We will now explore some of the mechanisms through which various pathologies can be sustained from freediving. At the outset, we delve into some of the principles that underpin these pathologies.

Boyle's law and barotrauma

Barotrauma refers to an injury sustained as a result of changes in pressure exerted on the body’s soft tissue. Boyle’s law states that there is an inverse relationship between the pressure and volume of a gas:

Pressure = (1/volume)

In freediving, the diver takes a single breath of air at the surface, where the atmospheric pressure is 1bar. With each 10m incremental increase in dive distance from the surface, the pressure increases by 1bar. Thus at 10m, the diver experiences 2bar of pressure, and at 40m, the diver experiences 5bar of pressure. These underwater pressure changes affect the volume of air-filled spaces within the body: sinuses, lungs, and ears. To counteract the adverse effects of this volume change, a diver must "equalise" as they descend to prevent barotrauma to the ears and sinuses. Equalising refers to the process of manually changing the pressure between the inside of the ear and the underwater environment: "popping your ears". Divers may equalise by using techniques such as the Valsalva or Frenzel manoeuvres [6].

Mammalian diving reflex

The mammalian diving reflex (MDR) is a collection of short-term physiological changes that occur in mammals upon diving into cold water. These changes have been observed in all species, which allow for oxygen conservation and subsequently increase the length of time they can remain submerged. These changes are summarised into a triad: bradycardia, apnoea, and increased peripheral vascular resistance. Splanchnic contraction, bradycardia, peripheral vasoconstriction, central vasodilation, and blood shift to the lungs all contribute to creating this physiological state [7].

Glossopharyngeal insufflation

More experienced and competition divers sometimes employ a technique called glossopharyngeal insufflation, also known as lung packing. This strategy increases the air volume in the lungs, increasing the length of time divers can stay underwater. However, glossopharyngeal insufflation has been shown to exacerbate physiological changes sustained while diving and can increase the risk of experiencing an adverse event from a dive [8].

Urge to breathe

For most people, the ‘urge to breathe’ is controlled by peripheral chemoreceptors that detect slight changes to blood pH caused by rising carbon dioxide levels. Many divers rely on this "urge to breathe" to determine when they should return to the surface to prevent loss of consciousness underwater. Inexperienced or untrained divers frequently hyperventilate before diving to increase their blood oxygen levels. This method is dangerous as hyperventilation causes them to expel large volumes of carbon dioxide, lowering their pre-dive carbon dioxide levels. They, therefore, experience the "urge to breathe" much later in the dive, which may lead to an underwater blackout. This mostly happens because the diver has depleted their oxygen stores before reaching the blood pH threshold to trigger the "urge to breathe", which would have alerted them to return to the surface.

Decompression illness

Decompression illness is the broad term for bubble-related pathology, well documented in scuba diving. Decompression illness occurs when inert gases within the circulatory system form bubbles when subjected to rises in pressure. These bubbles may lead to the formation of embolisms and subsequent tissue damage. Freediving was previously thought to be immune to this problem since the diver is not rebreathing at different pressures like in scuba diving. However, cases of decompression illness have been reported among divers undertaking multiple extended dives or practising glossopharyngeal insufflation [9].

Methods

A literature search was conducted on PubMed® to explore the range of injuries sustained in the sport of freediving. The search comprised the following key terms: ‘free-diving’, ‘breath-hold diving’ and ‘injuries’. The key terms ‘free-diving’ and ‘breath-hold diving’ were combined using the Boolean logic operator ‘OR’ to encompass studies using either definition of the sport. The key term ‘injuries’ was added using the Boolean logic operator ‘AND’ to limit the search to studies relevant to injuries incurred due to diving. Articles were limited to studies on humans and full-text articles. The initial search yielded 143 results. Abstracts and full articles were screened to eliminate studies that did not explore injuries or fatalities relevant to freediving. This screening method brought the number of articles down to 40 eligible studies for inclusion in our literature review.

Results

Injuries described in the literature were found in the following bodily systems: ophthalmological, ear, nose and throat (ENT), pulmonary, and neurological, and within each system, the mechanisms through which fatalities have occurred are explored here. The range of published pathologies is summarised in Table 1 below.

**Table 1:** Examples of the range of published pathologies sustained from freediving

Ophthalmological	ENT	Pulmonary	Neurological	Fatalities
Barotraumatic orbital emphysema	Inner-ear barotrauma	Pulmonary barotrauma	Decompression sickness	Apnoea hypoxia
Retinal vein occlusion	Perilymphatic fistulae	Alveolar haemorrhage	Cerebral infarcts	Autonomic conflict
	Exostoses	Pneumomediastinum	Brain injury	Cardiac arrhythmia
			Transient neurological injury	
			Non-traumatic spinal epidural haematoma	

Ophthalmological

Pressure-related injuries can affect the eye due to their proximity to the sinuses. For example, one case study described barotraumatic orbital emphysema with rhinogenic origin observed in a breath-hold diver [10]. The pressure changes experienced following multiple dives can also lead to the development of gas formation within vessels with a similar mechanism to decompression illness. One case study explored the retinal vein occlusion following an hour-long period of multiple breath-hold dives to a depth of 8m [11].

ENT

Barotrauma secondary to extreme pressures is well documented [12,13]. To avoid this phenomenon, freedivers continually equalise as they descend. Another pressure-related condition is perilymphatic fistulae: bubble-induced inner-ear damage leads to symptoms of hearing loss, dizziness, and tinnitus [14]. The literature also explored chronic changes related to freediving, with several studies investigating the prevalence of exostoses in experienced freedivers [15].

Pulmonary

Many studies in the literature review explored pulmonary pathology caused by pressures exerted on the lungs during freediving [16,17]. The most common mechanism described in the literature is referred to as pulmonary barotrauma leading to alveolar haemorrhage and presentation with haemoptysis [18-20]. The literature also described a case where glossopharyngeal insufflation caused pulmonary barotrauma and another case where repeated dives caused pneumomediastinum [21,22].

Neurological

The literature reports multiple cases of decompression sickness, thought to be caused by numerous recurrent or deep dives [23,24]. Brain injury, cerebral infarcts, and arterial gas emboli have all been reported as being caused by breath-hold diving [25-27]. Other published pressure-related pathology includes non-traumatic spinal epidural haematoma, transient neurological disorder, and mid-short-term memory impairments [18,28,29].

Fatalities

Fatalities were reported from several causes: drowning following an underwater blackout due to glossopharyngeal packing and apnoea hypoxia due to hyperventilation [30-32]. Reports of autonomic conflict leading to death have also been published. In these circumstances, the diver’s body initiated sympathetic stimulation in response to immersion in cold water. The body’s shock response produced parasympathetic-driven bradycardia, likely leading to cardiac arrhythmia [33].

## Conclusions

As is frequently the case in medicine, moderation reaps benefits, while excess may lead to adverse consequences. We presented the case report of a patient with a free-diving-induced pneumothorax and a pertinent literature review to explore the physiological changes incurred during freediving and explain the potential mechanisms of injury or fatality. Our findings highlight the range of pathologies associated with freediving, which divers are at higher risk of sustaining with poor breath-holding techniques or inadequate training. Therefore, this study supports the importance of receiving pre-dive medical checks to evaluate specific bodily systems that may be particularly vulnerable; however, it is important to note that although a rigorous pre-dive medical check can improve safety, it cannot remove all of the risks.

A thorough discussion of precautions is beyond the scope of this article; however, this is one of the areas where further research is warranted. Further exploration into the incidence of injuries associated with freediving would also give an indication of the prevalence of adverse events. However, given the relative scarcity of people who engage in this sport, it may be challenging to design an observational study with adequate statistical power to address this research question. Furthermore, this field would benefit from further research into the potential benefits that the sport can bring to individuals and communities so that an informed evaluation of the risks and benefits can be undertaken.
